# Virulent Strain of Hepatitis E Virus Genotype 3, Japan

**DOI:** 10.3201/eid1505.081100

**Published:** 2009-05

**Authors:** Kazuaki Takahashi, Hiroaki Okamoto, Natsumi Abe, Manri Kawakami, Hiroyuki Matsuda, Satoshi Mochida, Hiroshi Sakugawa, Yoshiki Suginoshita, Seishiro Watanabe, Kazuhide Yamamoto, Yuzo Miyakawa, Shunji Mishiro

**Affiliations:** Toshiba General Hospital, Tokyo, Japan (K. Takahashi, N. Abe, S. Mishiro); Jichi Medical University School of Medicine, Tochigi, Japan (H. Okamoto); Kurashiki Medical Center, Okayama, Japan (M. Kawakami); Matsuda Naika Clinic, Tottori, Japan (H. Matsuda); Saitama Medical University, Saitama, Japan (S. Mochida); Heart-Life Hospital, Okinawa, Japan (H. Sakugawa); Kyoto University Graduate School of Medicine, Kyoto, Japan (Y. Suginoshita); Kagawa Prefectural Central Hospital, Kagawa, Japan (S. Watanabe); Okayama University Graduate School of Medicine, Dentistry and Pharmaceutical Sciences, Okayama (K. Yamamoto); Miyakawa Memorial Research Foundation, Tokyo (Y. Miyakawa)

**Keywords:** Zoonoses, viruses, hepatitis E virus, acute hepatitis, helicase domain, JIO strain, severe hepatitis, swJ19 strain, research

## Abstract

Hepatitis E virus (HEV) genotype 3, which usually causes asymptomatic infection in Japan, induced severe hepatitis in 8 patients. To better understand genetic features of HEV associated with increased virulence, we determined the complete or near-complete nucleotide sequences of HEV from these 8 patients and from 5 swine infected with genotype 3 strain swJ19. Phylogenetic analysis showed that the isolates from the 8 patients and the 5 swine grouped separately from the other genotype 3 isolates to create a unique cluster, designated JIO. The human JIO-related viruses encoded 18 amino acids different from those of the other HEV genotype 3 strains. One substitution common to almost all human HEV strains in the JIO cluster was located in the helicase domain (V239A) and may be associated with increased virulence. A zoonotic origin of JIO-related viruses is suspected because the isolates from the 5 swine also possessed the signature V239A substitution in helicase.

## Abstract 2

Virulence may be associated with mutation of the helicase domain (V239A), and source of the human infection may be swine.

Hepatitis E virus (HEV) infection is relatively common. Anti-HEV antibodies are found in 10%–20% of the general population in Japan and most Asian countries (*1*,*2*); however, only a small fraction of these infections induce overt hepatitis. Although the mechanisms underlying induction of liver damage by HEV have not been well characterized, HEV genotypes seem to have distinct disease-inducing potential. HEV sequences have been classified into 4 genotypes (*3*). Genotype 1 consists of epidemic strains in developing countries of Asia and Africa. Genotype 2 is represented by the prototype sequences from an epidemic in Mexico, which have also recently been detected in Africa. Genotypes 3 and 4 are distributed worldwide and have been implicated in sporadic cases of acute hepatitis E in humans and domestic pigs. HEV genotypes 3 and 4 are found in Japan, but fulminant or severe acute hepatitis develops more frequently in persons infected with genotype 4 (*4*–*6*). The severity of liver disease may therefore be influenced by the HEV genotype with which the patient is infected as well as host factors such as age, gender, and pregnancy status.

In 1997, we identified a strain of HEV from a patient in Japan who had acute hepatitis (designated JIO) that clustered with genotype 3 sequences. From 2004 through 2006, JIO-related viruses were isolated from 7 additional patients who had acute or severe hepatitis. To better understand genetic features of HEV associated with severe hepatitis, we compared the complete or near-complete sequence of JIO isolates from these 8 patients with other well-characterized genotype 3 and 4 isolates. To determine whether these human genotype 3 sequences were zoonotic in origin, we sequenced full-length viral genomes from 5 swine infected with the swJ19 strain of HEV. These 5 animals were part of a larger outbreak of HEV infection that occurred in swine at a single farm in southern Japan during 2000–2002. The GenBank/EMBL/DDBJ accession numbers for nucleotide sequences of HEV isolates are AB291951–7/AB291960 (for the human isolates) and AB443623-7 (for the swine isolates).

## Methods

**Figure 1:**
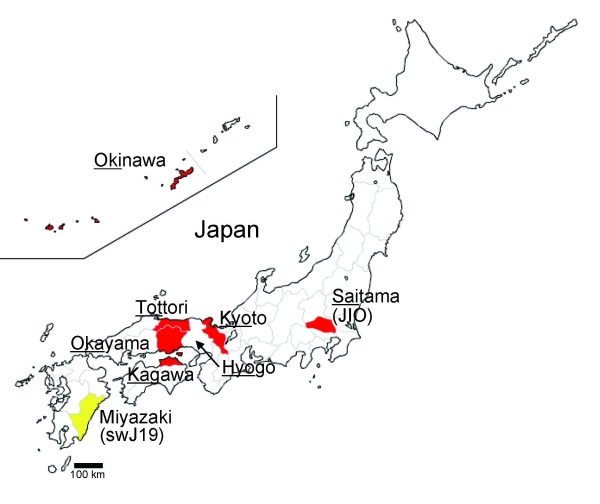
Map of Japan showing prefectures where human cases of hepatitis E virus have been found. Underlining indicates part of prefecture name included in isolate name; yellow indicates cases in swine; red indicates cases in humans.

We enrolled 8 patients who were infected with HEV genotype 3 and had clinical features of hepatitis (Table 1). A zoonotic source of HEV infection was identified for 3 of these patients: pig liver for patient 4, pig meat for patient 6, and wild boar meat for patient 7. Prothrombin time, a surrogate marker of hepatic insufficiency, averaged 63.9% (± 29.1%) of the reference range among the 8 HEV genotype 3–infected patients. Hepatitis was particularly severe in patients 3, 5, 7, and 8; at the peak of disease, prothrombin times for these patients ranged from 27% to 46% of the reference range. These sporadic HEV cases were not clustered geographically; they were distributed across several regions of Japan, including southern (Okinawa) and northern (Saitama) prefectures (Figure 1). Informed consent was obtained from all patients after the nature and purpose of the study was explained to them.

**Table 1:** Profiles of 8 patients infected with hepatitis E virus, JIO strain, Japan*

Patient no.	Age, y/sex	Residence	Month of disease onset	Diagnosis	Nadir PT, %	Presumed route of transmission	Isolate name
1	50/M	Saitama	1997 Apr	Acute hepatitis	100	Unknown	JIO-Sai97L
2	76/M	Tottori	2004 Jan	Acute hepatitis	92	Unknown	JYM-Tot04L
3	63/M	Okinawa	2004 May	Acute hepatitis	46	Unknown	JYU-Oki04L
4	71/F	Okayama	2004 Dec	Acute hepatitis	75	Pig liver	JSS-Oka04L
5	65/M	Tottori	2005 Jun	Acute severe hepatitis	34	Unknown	JIY-Tot05L
6	78/M	Okinawa	2005 Jul	Acute hepatitis	92	Pig meat	JSO-Oki05L
7	63/M	Kagawa	2006 Mar	Acute hepatitis	45†	Wild boar meat	JTK-Kag06C
8	79/M	Kyoto	2006 Sep	Fulminant hepatitis	27	Unknown	JSW-Kyo-FH06L

*PT, prothrombin time. †Only 1 determination was made.

To assess possible zoonotic origins of these human infections, we sequenced HEV strain swJ19 isolates from 5 of 11 swine with previously documented infections (*7*). These animals had been raised commercially at a farm in the southern part of Miyazaki Prefecture where HEV infections were detected during 2000–2002. All animals received humane care, and the study was approved by the institutional review committee of Toshiba General Hospital, Tokyo, Japan.

To determine whether infections could be linked to a common genotype 3 virus, we compared the genetic structure and sequence homology of 8 human and 5 swine HEV strains. The entire or near-complete nucleotide sequences of the 8 JIO strain isolates from the human patients and the swJ19 strain isolates from the 5 swine were determined by a method reported previously (*8*,*9*), with some modifications. In brief, nucleic acids were extracted from serum with the QIAamp MinElute Virus Spin Kit (QIAGEN GmbH, Hilden, Germany). HEV RNA genomes were reverse transcribed, and cDNA was amplified by PCR with primers specific for 23 overlapping regions of the HEV genome. Reverse transcription and first-round PCR were conducted by using the SuperScript III One-Step RT-PCR System (Invitrogen Corporation, Carlsbad, CA, USA); second-round PCR was conducted with the Platinum Taq DNA polymerase (Invitrogen). The 5′- and 3′-terminal sequences were amplified by using the SMART RACE cDNA Amplification Kit (Clontech Laboratories Inc., Mountain View, CA, USA) and Oligo (dt)20 Primer (Invitrogen), respectively. The sequences enriched in G-C were amplified with the TaKaRa LA Taq in GC Buffer (TaKaRa Shuzo Co. Ltd., Shiga, Japan). The sequences not amplifiable by the above PCR methods were subjected to PCR with primers deduced from adjacent 5′ and 3′ sequences. The final products were sequenced in the 377 DNA Sequencer with use of the BigDye Terminator v1.1 Cycle Sequencing Kit (Applied Biosystems, Foster City, CA, USA). Genetic analyses of HEV sequences were conducted by the unweighted pair-grouping method with arithmetic means by using computer software GENTYX-MAC Version 13 (Genetyx Corporation, Tokyo, Japan).

## Results

The prototypical isolate, JIO-Sai97L, had a genome length of 7,215 nt that contained a 5′ untranslated region (UTR), 3 open reading frames (ORFs), a 3′ UTR, and a poly-A tail. Lengths of HEV genomes from 6 other patients (JYM-Tot04L, JYU-Oki04L, JSS-Oka04L, JIY-Tot05L, JSO-Oki05L and JSW-Kyo-FH05L) were identical to that of JIO-Sai97L. An exception was the HEV isolate JTK-Kag06C from patient 7, which was slightly longer (7,236 nt). The 5 HEV isolates from swine (swJ19-1, swJ19-2, swJ19-5, swJ19-7, and swJ19-8) had genomes of 7,210 nt. The 3 ORFs of all swine and human HEV genomes had identical protein coding capacity. HEV isolates from all human patients had 97.9%–98.6% sequence homology with the prototypical JIO-Sai97L strain from patient 1. The 5 swine swJ19 isolates had 98.3%–99.9% sequence homology when compared with each other and 98.0%–99.8% homology when compared with the JIO strain from human patients.

Comparison of nucleotide sequences of the 13 human and swine HEV isolates in this study with those of published HEV genotype 3 sequences showed that the 13 complete and near-complete sequences described in this study closely matched those of 2 well-characterized genotype 3 viruses: JRA1 (89.4%–89.7% nucleotide identity) and swJ570 (88.9%–89.0% nucleotide identity). The 13 human and swine genotype 3 isolates displayed weak homology with other HEV genotypes. The B1 isolate of genotype 1 (GenBank accession no. M73218) was only 74.1%–74.7% similar to these genotype 3 viruses, the M1 isolate of genotype 2 (accession no. M74506) was only 73.6%–74.0% similar, and the T1 isolate of genotype 4 (accession no. AJ272108) was only 75.6%–76.0% similar.

**Figure 2:**
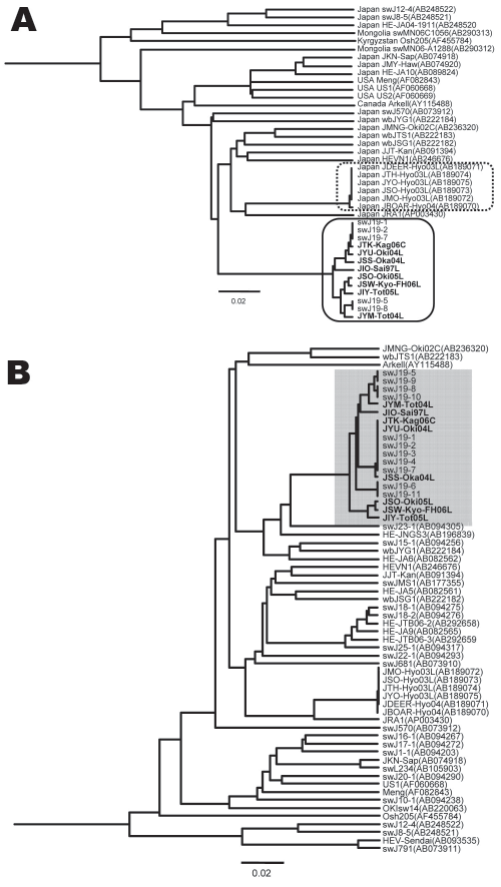
A) Phylogenetic tree (unweighted pair-grouping method with arithmetic means) constructed on the complete or near-complete nucleotide sequences of hepatitis E virus (HEV) genotype 3 isolates. Clustering of nucleotide sequences of 8 human patients infected with JIO strain of HEV and 5 swine infected with swJ19 strain of HEV is boxed by a solid line. Another clustering of local genotype 3 isolates from Hyogo Prefecture, Japan, is boxed by a dotted line. B) Phylogenetic tree (unweighted pair-grouping method with arithmetic means) constructed on a partial sequence of 412 nt in open reading frame (ORF) 2 (nt 5994-6405 of the US2 genome) of HEV genotype 3. Partial nucleotide sequences of 8 human JIO and 11 swine HEV swJI9 isolates (accession nos. AB094279–AB094289) are shown (shading). Analyses of full sequences of 5 of these 11 swine isolates were performed (swJ19-1, swJ19-2, swJ19-5, swJ19-7, and swJ19-8). Scale bars indicate nucleotide substitutions per site; **boldface** indicates isolates from humans.

Using the 13 complete or near-complete genomic sequences of HEV genotype 3 isolates described in this study (Figure 2), we constructed a phylogenetic tree. HEV sequences from the 8 patients (JTK-Kag06C, JYU-Oki04L, JSS-Oka04L, JIO-Sai97L, JSO-Oki05L, JSW-Kyo-FH06L, JIY-Tot05L, JYM-Tot04L) clustered on a branch separate from the other genotype 3 sequences, forming a distinct grouping related to the prototypical JIO strain. The swJ19 HEV sequences from the 5 swine (swJ19-1, swJ19-2, swJ19-7, swJ19-5, and swJ19-8) clustered closely with the JIO-related viruses from the human patients, indicating that the human and swine HEV isolates were highly similar (Figure 2, panel A). Another 18 swine isolates, from farms other than the 1 involved in the swJ19 outbreak, were phylogenetically distinct from those of the outbreak farms (Figure 2, panel B).

Another genotype 3 cluster was formed by 6 isolates from Hyogo Prefecture in western Japan (Figure 2, panel A). In this cluster were 5 HEV isolates from persons in whom hepatitis developed after they ate uncooked deer meat (*10*) and from serum from a local boar and a deer (*11*). Unlike the JIO-related viruses, which were broadly distributed from the most southern to northern Japanese prefectures, HEV strains responsible for the infections in Hyogo Prefecture were not commonly found in other parts of the country. Broad distribution of the JIO-related viruses seems to be unique in HEV epidemiology. In 2 (25%) of these 8 patients, pig liver or meat had been implicated in HEV infection.

Comparison of the 13 JIO-related viruses (Figure 2, panel A) with the other genotype 3 strains also showed 18 aa differences: 12 in ORF1, 3 in ORF2, and 3 in ORF3 (Table 2). Three mutations in the JIO strain were characteristic of genotype 4 viruses, which are typically more pathogenic than other HEV genotypes. ORF1 differences were found at amino acids 605 (serine to proline, S605P), 978 (isoleucine to valine, I978V), and 1213 (valine to alanine, V1213A). The V1213A substitution is potentially most relevant because it was not found in the prototypical isolate from patient 1 (JIO), who had mild clinical disease when infected in 1997, but was present in highly related isolates from the other 7 patients who had more severe hepatitis during 2004–2006. V1213A in ORF1 corresponds to V239A of the helicase domain, and its surrounding sequences were well conserved in HEV isolates of genotypes 3 and 4 (Appendix Figure). Because V239A is common in genotype 4 isolates, we analyzed genomes of the genotype 3 JIO-related viruses for evidence of intergenotypic recombination. Comparison of 28 genotype 4 sequences with those of the JIO-related isolates showed no obvious signs of recombination (data not shown), which suggests that the V293A substitution arose independently in this genetically unique cluster of genotype 3 viruses. Notably, all 5 isolates recovered from swine on the Miyazaki Prefecture farm during the outbreak of 2000–2002 possessed the V239A substitution.

**Table 2:** Amino acid residues in 8 human and 5 swine hepatitis E virus isolates compared with those in the other genotype 3 isolates*

Amino acid position†	Conserved in genotype 3	Human no.									Swine no.					Conserved in genotype 4
		1	2	3	4	5	6	7	8		1	2	3	4	5	
ORF1																
154	A	A	T	A	A	T	T	A	T		A	A	T	A	T	T
547	R	Q	Q	Q	Q	Q	Q	Q	Q		Q	Q	Q	Q	Q	R
598	R	Q	Q	Q	Q	Q	Q	Q	Q		Q	Q	Q	Q	Q	K
**605**	S	P	P	P	P	P	P	P	P		P	P	P	P	P	P
721	A	T	T	T	T	T	T	T	T		T	T	T	T	T	A
807	A	S	S	S	S	S	S	S	S		S	S	S	S	S	A
**978**	I	V	V	V	V	V	V	V	V		V	V	V	V	V	V
979	S	K	K	K	K	K	K	K	K		K	K	K	K	K	E
1135	I	T	T	T	T	T	T	T	T		T	T	T	T	T	V
**1213‡**	V	V	A	A	A	A	A	A	A		A	A	A	A	A	A
1246	Q	H	H	H	H	H	H	H	H		H	H	H	H	H	D
1469	C	S	S	S	S	S	S	S	S		S	S	S	S	S	C
ORF2																
98	P	S	S	P	P	S	S	P	P		P	P	S	P	S	A
113	V/I	T	T	T	T	T	T	T	T		T	T	T	T	T	V
660	S	S	S	S	F	F	F	S	F		S	S	S	S	S	Y
ORF3																
91	S	N	N	N	N	N	N	N	N		N	N	N	N	N	S
97	A	A	V	V	V	V	V	V	V		V	V	V	V	V	A
98	P	Q	Q	Q	Q	Q	Q	Q	Q		Q	Q	Q	Q	Q	P

*Eighteen amino acids of 8 human isolates (JIO strain) and 5 swine isolates (swJ19 strain) not shared by other genotype 3 isolates. The 3 at positions 605, 978, and 1213 (**boldface**) were the same as the corresponding residues in genotype 4 isolates. †Corresponds to the position in hepatitis E virus (HEV)-US2 (GenBank/EMBL/DDBJ accession no, AF060669) (*12*). ‡V1213A in the open reading frame (ORF) 1 polyprotein corresponds to V239A in the HEV-US2 genotype 3 isolate helicase domain within ORF1 (online Appendix Figure.

## Discussion

Circumstantial evidence indicates that HEV genotype influences the severity of liver disease. Remarkably, HEV seroprevalence studies in Egypt found no clinical illness in any person, including pregnant women, although most (67.7%–84.3%) had been exposed to HEV genotype 3 (*13**,**14*). In contrast, results of a survey of 254 patients with HEV infection in Japan showed hepatitis associated with genotype 4 to be more severe than that associated with genotype 3 (*4*). Our results showed a clustering of 8 HEV isolates of JIO strain, genotype 3, recovered from patients with clinical hepatitis.

Despite the high disease-inducing activity of the HEV JIO strain, the 8 patients infected with this strain were distributed widely over Japan. This distribution is at odds with a local cluster of genotype 3 infections restricted to persons with hepatitis and to wild animals living in Hyogo Prefecture, Japan (Figure 2, panel A) (*11*). Wide regional distribution has also been reported for some HEV genotype 4 isolates (*15*). Why JIO strains caused more severe hepatitis than might be expected for a genotype 3 virus is not clear, but the reason may depend on the magnitude of virus replication. Alternatively, recombination between divergent HEV strains (*16*) may have played a role. This possibility prompted us to look for any recombination of JIO strains with genotype 4 strains that cause severe hepatitis in Japan. However, we found no evidence of recombination between the JIO strain of genotype 3 HEV with which the 8 persons were infected and 28 isolates of genotype 4 retrieved from the public and our own databases. The 18 aa substitutions were unique to the 8 human JIO and 5 swine sw19 isolates and not present in other genotype 3 viruses. Three differences in ORF1 (S605P, I978V, and V1213A) were common in wild type genotype 4 but not in genotype 3 isolates (Table 2). Because S605P and I978V are located in an ORF1 region that has high sequence divergence, they are unlikely to be responsible for an enhanced disease-inducing capacity. In contrast, V1213A changes at amino acid 239 of helicase, an enzyme capable of enhancing the efficiency of viral replication (*17*), were detected in 7 of the 8 patients (Appendix Figure). Indeed, the helicase region of the prototypical JIO-Sai97L isolated in 1997 did not contain this amino acid polymorphism. Remarkably, all 5 swine isolates recovered in Miyazaki Prefecture during 2000–2002 belonged to the JIO strain and possessed V1213A (*hel*V239A). Taken together, the evidence strongly suggests a zoonotic origin for the 8 human HEV infections with JIO-related viruses.

Experimental and circumstantial evidence suggests that *hel*V239A may have enhanced the helicase activity of the genotype 3 JIO strain to levels comparable with those of the more pathogenic genotype 4 viruses. However, the role of *hel*V239A in enhancing helicase activity should be evaluated in vitro in future studies; its role in inducing hepatitis is yet to be confirmed. In addition, the effect of other mutations of JIO strains need to be fully explored before a conclusion can be drawn regarding the hepatitis-inducing capacities of this strain of HEV.

Findings from this study have public health implications. Because farm swine constitute a melting pot for generating various HEV mutants, at least in Japan where virtually all swine become infected with HEV within 4 months of birth, it is conceivable that virulent HEV mutant(s) arise on pig farms. Such occurrence has been described for influenza, for which point mutations are associated with increased virulence (*18**,**19*); for example, mutant influenza viruses that arose on chicken farms in Hong Kong in 1997 were transmitted to humans and had fatal consequences (*20**,**21*). In addition, although a vaccine against HEV has recently been developed (*22*), a vaccination strategy for humans and animals has yet to be defined. The results of our study indicate that selective vaccination of farm swine bearing HEV isolates of high virulence, such as those of the JIO strain in Miyazaki Prefecture, should be recommended to decrease the incidence of fulminant or severe acute hepatitis E in Japan and elsewhere in the world.

## Supplementary Material

Appendix Figure Alignment of a partial C-terminal amino acid sequence of the helicase domain of hepatitis E virus (HEV) open reading frame (ORF) 1 across genotype 3 and 4 isolates. A bracket indicates 8 isolates of the JIO strain HEV genotype 3 and 5 isolates of swJ19 strain. Conversions in helV239A are shown by linear box. Partial sequences of ORF1 (aa positions 1105-1226 of HEV-US2) of human and swine isolates of genotype 3 were compared.
