# Supplementary figures and images for: Virulent Strain of Hepatitis E Virus Genotype 3, Japan
Source: Emerg Infect Dis. 2009 May;15(5):704–9. doi: 10.3201/eid1505.081100 (PMC2687009; doi:10.3201/eid1505.081100)

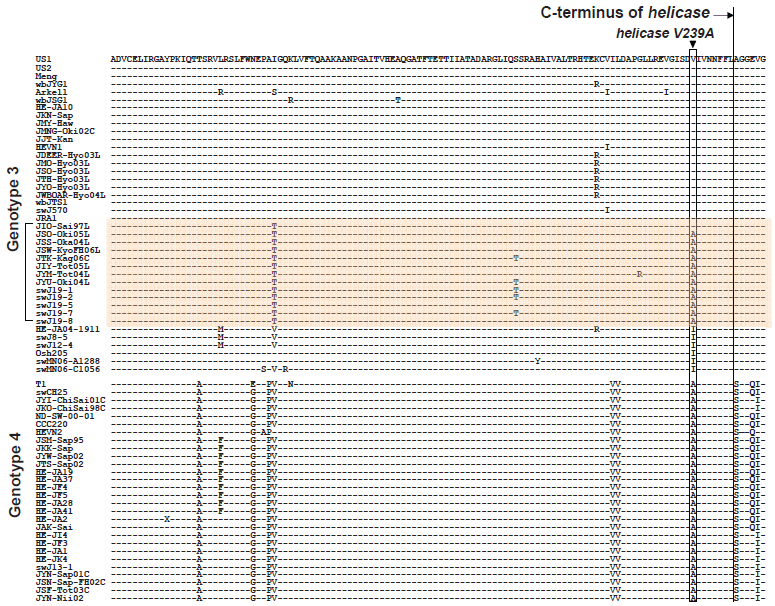

Supplement: Appendix Figure — Alignment of a partial C-terminal amino acid sequence of the helicase domain of hepatitis E virus (HEV) open reading frame (ORF) 1 across genotype 3 and 4 isolates. A bracket indicates 8 isolates of the JIO strain HEV genotype 3 and 5 isolates of swJ19 strain. Conversions in helV239A are shown by linear box. Partial sequences of ORF1 (aa positions 1105-1226 of HEV-US2) of human and swine isolates of genotype 3 were compared. [file 08-1100_app-s1.gif]
